# Association of type 2 diabetes with reoperation, adverse events and mortality after hip and knee replacement: a Swedish register-based study including 109 938 hip and 80 897 knee replacements

**DOI:** 10.1136/bmjopen-2024-096717

**Published:** 2025-09-28

**Authors:** Johanna Vinblad, Erik Bülow, Fredrik Nyberg, Katarina Eeg-Olofsson, Annette W-Dahl, Gunilla Limbäck, Martin Englund, Allan Abbott, Andrea Dell’Isola, Ola Rolfson

**Affiliations:** 1 Centre of Registers Vastra Gotaland, Goteborg, Sweden; 2 Department of Orthopaedics, Institute of Clinical Sciences, Sahlgrenska Academy, University of Gothenburg, Goteborg, Sweden; 3 School of Public Health and Community Medicine, Institute of Medicine, Sahlgrenska Academy, University of Gothenburg, Goteborg, Sweden; 4 Department of Molecular and Clinical Medicine, Institute of Medicine, Sahlgrenska Academy, University of Gothenburg, Goteborg, Sweden; 5 Department of Medicine, Sahlgrenska University Hospital, Goteborg, Sweden; 6 Department of Orthopedics, Faculty of Medicine, Clinical Science Lund, Lund University, Lund, Sweden; 7 Faculty of Medicine, Department of Clinical Sciences Lund, Orthopedics, Clinical Epidemiology Unit, Lund University, Lund, Sweden; 8 Department of Occupational and Physical Therapy, Sahlgrenska University Hospital, Goteborg, Sweden; 9 Unit of Physiotherapy, Department of Health, Medicine and Caring Sciences, Linköping University, Linkoping, Sweden; 10 Department of Orthopaedics, Linköping University Hospital, Linkoping, Sweden; 11 Department of Ortopaedics, Sahlgrenska University Hospital, Goteborg, Sweden

**Keywords:** Mortality, Hip, Knee, Diabetes Mellitus, Type 2, REGISTRIES, Adverse events

## Abstract

**Abstract:**

**Objective:**

Type 2 diabetes mellitus (T2DM) and osteoarthritis (OA) are globally prevalent chronic diseases that affect millions of individuals in ageing populations. Hip and knee replacements are well established and effective treatments in patients suffering from end-stage OA. Understanding how T2DM influences the outcomes of these surgeries is important for optimising patient care and improving surgical results. This study aimed to explore the association of T2DM with reoperation (regardless of the reason), adverse events (AEs) and mortality after primary hip and knee replacement surgery.

**Design:**

Observational study based on prospectively collected registry data analysed retrospectively.

**Setting and participants:**

Data from several Swedish national quality registers and health data registers were used to create a study database. 109 938 and 80 897 primary hip and knee replacements due to OA, performed between 2008 and 2019 (hip) and 2009 and 2018 (knee), were included in the study.

**Outcome measures:**

The risk of complications, such as reoperation, AEs and mortality, was investigated by estimating HRs using Cox regression, and OR using logistic regression, unadjusted and adjusted for confounding factors, such as patient characteristics, socioeconomic status and comorbidities, and mediators, such as surgical factors.

**Results:**

Adjusted multivariable Cox-regression analysis showed no T2DM-associated risk of reoperation after hip or knee replacement, adjusted HR 1.10 (95% CI 0.99 to 1.23) and 1.09 (95% CI 0.96 to 1.24), respectively, while T2DM was associated with increased risk of death after hip and knee replacement, adjusted HR 1.40 (95% CI 1.34 to 1.47) and 1.38 (95% CI 1.31 to 1.45). Adjusted logistic regression analysis showed T2DM-associated increase of reoperation within 90 days (OR 1.23 (95% CI 1.05 to 1.43)) and increased mortality within 90 days (OR 1.42 (95% CI 1.01 to 1.95)) following hip replacement; however, this was not the case after knee replacement, OR 1.08 (95% CI 0.85 to 1.36) for reoperation and OR 1.29 (95% CI 0.84 to 1.94) for mortality. Several factors closely linked with T2DM, such as body-mass index and comorbidities, were identified as important when assessing risk of reoperation and mortality. Regarding AEs within 30 and 90 days, very slight but not statistically significant T2DM-associated increases were seen after either hip replacement, OR 1.01 (95% CI 0.91 to 1.11) and 1.07 (95% CI 0.98 to 1.16) or after knee replacement, OR 1.05 (95% CI 0.93 to 1.17) and 1.08 (95% CI 0.98 to 1.19).

**Conclusion:**

The observed risk of reoperation suggests that T2DM alone was not a strong justification to advise against hip or knee replacement in individuals with T2DM deemed eligible for joint replacement. The T2DM-associated increased mortality after hip and knee replacement is challenging to interpret, as T2DM itself without undergoing hip or knee replacement surgery is associated with increased mortality.

**Strengths and limitations of this study**

- This study had close to complete the coverage of the Swedish population.
- The outcomes were adjusted for confounding factors, for example, age, body-mass index, sex and comorbidities.
- Register-based studies are limited by the available variable and outcome options as well as patients from the data sources.
- As the study was based on Swedish data, the findings may not be fully generalisable to countries with different welfare systems.

## Introduction

 Diabetes exists in several forms, with type 2 diabetes mellitus (T2DM) being the most common. T2DM is characterised by insulin resistance and is often associated with obesity, age and other metabolic risk factors, such as hypertension and hyperlipidaemia. The global prevalence of diabetes has been steadily increasing, with an estimated 10.5% of the adult population affected in 2021.^1^ The global prevalence of osteoarthritis (OA) was 7.6% in 2020^2^ and is almost two times as high in the T2DM population compared with the non-diabetes population (NT2DM).^3^ OA is characterised by chronic pain and loss of mobility. A metabolic link between OA and diabetes has been suggested.^4^ The first-line intervention of OA encompasses patient education, exercise therapy and weight control.^5^ Joint replacement is considered for individuals with severe radiographic changes and intolerable pain, despite non-surgical treatments.^6^ The need for hip or knee replacement will grow as the population gets older and, therefore, it will, in the future, be even more critical to understand patient-specific risk factors related to complications, especially as complications are known to be associated with high costs for the healthcare system and reduce the quality of life.^7^,^9^

Previously published studies often identify diabetes to be associated with an increased risk for complications after hip or knee replacement.^10^,^15^ However, there are publications suggesting no significant association between T2DM and revision or adverse events.^16 17^ The definition of complication, size of studied cohort and definition of diabetes diagnosis varies between studies, adding to the complexity in interpreting the results. Lenguerrand *et al* suggest that poorer outcomes after total hip and knee replacement are associated more with obesity and other comorbidities rather than with diabetes.^17^

To address the inconsistency of previous literature, the current study aimed to assess the association between T2DM and complications, such as reoperation, adverse events and mortality after OA-related hip and knee replacement, using a close to complete coverage nationwide Swedish population data source.

## Methods

### Study design

This study was based on the Swedish OA and Diabetes project cohort. The cohort was constructed by linking data from multiple national registers in Sweden. Clinical information related to treatment and interventions associated with respective diagnoses was obtained from the Swedish Arthroplasty Register, the National Diabetes Register and the Swedish OA Register. Comorbidity data were extracted from the National Patient Register, which contains diagnoses from specialist inpatient and outpatient care, while the Prescribed Drug Register provided information on pharmacological treatments, used as proxies for comorbid conditions. Demographic variables were sourced from the Total Population Register, and socioeconomic indicators were retrieved from the Longitudinal Integrated Database for Health Insurance and Labour Market Studies (LISA). Finally, the Cause of Death Register from Statistics Sweden supplied data on mortality and causes of death. Statistics Sweden was responsible for linking the data. A detailed protocol for the project database and design has previously been published.^18^ The investigator in this study had full access to the study database. Patients or the public were not involved in the design, conduct, reporting or dissemination plans of this study.

### Study cohort inclusion

**Figure 1:**
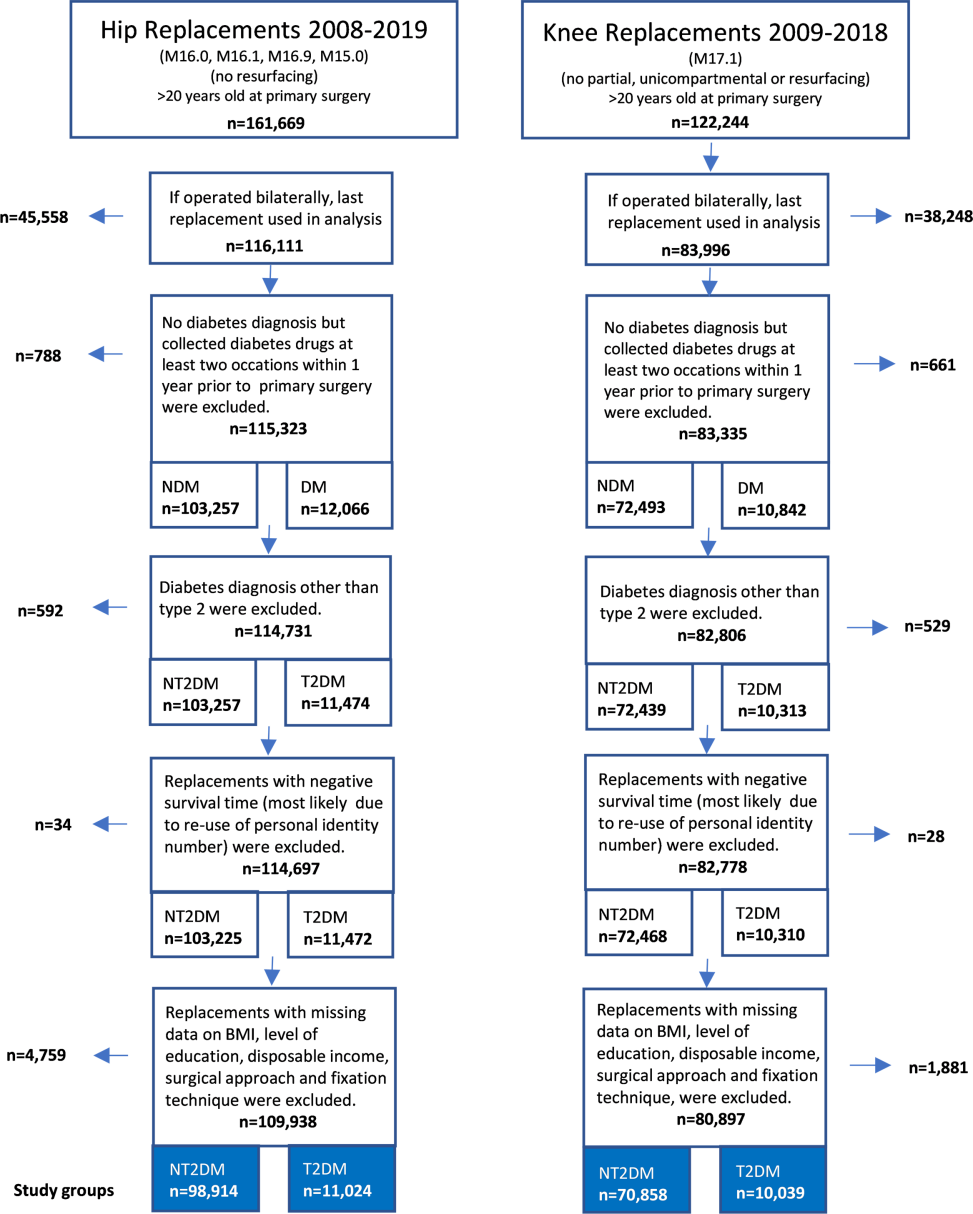
Cohort selection flowchart. BMI, body mass index; DM, diabetes mellitus; NDM, no diabetes mellitus; NT2DM, no diabetes population; T2DM, type 2 diabetes mellitus.

A flowchart describing the selection of the final study cohort in the current study is presented in figure 1. Individuals with registered primary total hip replacement between 2008 and 2019 or primary total knee replacement between 2009 and 2018 in the Swedish Arthroplasty Register due to OA (International Classification of Diseases version 10 (ICD-10) codes M16.0, M16.1, M16.9 or M15.0 and M17.1 for hip and knee, respectively) were included in the study. The difference in study period between hip and knee was due to data availability. To ensure confounding baseline data 18 months prior to surgery, individuals from 20 years of age at surgical date of primary hip or knee replacement were included. Further, to keep the cohort as homogenous and relevant as possible, hip resurfacing and partial knee replacements (unicompartmental, patellofemoral and resurfacing knee replacements) were excluded from the study cohort. Based on the findings from Bülow *et al,* if hips or knees were operated bilaterally during the study period, the first replacement surgery was excluded as the second replacement to a larger extent resembles unilateral total hip replacement.^19^ If the primary hip or knee replacement surgery was performed on both sides on the same day, the side used in the analysis was chosen at random by using the right side for even numbered subject identifiers in the linked data, and the left-side operation for uneven numbered. 45 558 out of 161 669 replacements were excluded due to bilateral hip replacements and 38 248 out of 122 244 due to bilateral knee replacements. In the NT2DM group, dispensed diabetes drugs (anatomic therapeutic chemical classification system (ATC) code beginning with A10 in the Swedish Prescribed Drug Register from the National Board of Health and Welfare) at least two times during the year prior to primary hip or knee replacement surgery excluded 788 hip replacements and 661 knee replacements, as it was unclear if the individuals were diagnosed with T2DM or not. As different types of diabetes are associated with different disease mechanisms and to fully focus on T2DM, 592 hip and 529 knee replacements were excluded due to other types of diabetes. 34 hip and 28 knee replacements with negative patient survival time, probably due to reused personal identity^20^ numbers or inaccurate registrations, were also excluded. Finally, patients with hip and knee replacements with missing data for potential confounders and mediators, such as body-mass index (BMI), level of education and disposable income, incision and surgical fixation technique, were excluded, 4759 (4.1 % of 114 697) and 1881 (2.3% of 82 778) replacements for hip and knee, respectively. This resulted in the final study cohort encompassing 109 938 (98 914 NT2DM and 11 024 T2DM) and 80 897 (70 858 NT2DM and 10 039 T2DM) hip and knee replacements, respectively. Outcomes after hip or knee replacement were analysed separately. A total of 6493 individuals with both hip and knee replacement were included in both analyses.

### Exposure measures

T2DM is defined as a record of T2DM diagnosis in the Swedish National Diabetes Register (ICD-10 code E11) before primary hip or knee replacement.

### Outcome measures

The risk of reoperation for hip and knee replacement was followed until 31 December 2019 and 31 December 2018, respectively. Additionally, the risk of death (mortality) following both hip and knee replacement was followed until 31 December 2020, and adverse events were followed until 31 December 2019 for both joints. The length of follow-up time for the outcome measures was dependent on the data availability. The reoperation and mortality analysis was conducted using Cox regression. In addition, the risk of reoperation within 90 days, adverse events within 30 and 90 days and 90-day mortality were explored using logistic regression. For hip replacements, reoperation includes both revision (any procedure where any implant is exchanged or removed) and other open surgical procedures. Other procedures were not included in the definition of knee reoperation, just revisions. Knee revision is defined as any procedure where the implant is exchanged, removed or added (including arthrodesis and amputation above the knee).

The Swedish Arthroplasty Register’s definition of adverse event after total hip or knee replacement was used. The definition of adverse event is based on a selection of ICD-10 and Nordic Medico-Statistical Committee (NOMESCO) codes specifically relevant after hip and knee replacement.^21^ Codes for adverse events were obtained from the National Patient Register at the National Board of Health and Welfare. Due to the absence of adverse event diagnosis date for inpatient care in the National Patient Registry, the date of admission was used as a proxy for adverse event diagnosis date. Date of death was obtained from Statistics Sweden’s Total Population Register.

### Confounders and mediators

Age and BMI at primary replacement surgery, sex, year of primary surgery, level of education, individual disposable income level and comorbidity were identified as confounding factors and adjusted for in the analysis. Side of surgery, fixation technique and incision (incision only available for hip replacement) were explored as mediators. Information on age and BMI at the time of primary surgery, sex, year of primary surgery, side of primary surgery, fixation technique and incision at primary surgery was obtained from the Swedish Arthroplasty Register. Level of education according to the Swedish education nomenclature SUN 2000 and individual disposable income level (total income received and measured from registers, including allowances minus taxes paid) the year prior to surgery were obtained from the LISA at the National Board of Health and Welfare. To adjust for inflation, the disposable income was adjusted with a study-specific index where the disposable mean income for the cohort in the reference year 2019 was divided by the disposable mean income for the year of interest. The created index was then applied to the disposable income 1 year prior to index surgery. To calculate the comorbidity index RX risk score, the ATC codes for prescribed and collected drugs 1 year prior to primary surgery were obtained from the Prescribed Drug Register at the National Board of Health and Welfare. The index was calculated as described by Pratt *et al*^22^ with the modification that diabetes-related ATC codes (A10AA01–A10BX99) were excluded. RX risk score captures a wider set of comorbidities compared with other known indices, such as Elixhauser and Charlson, as they are limited to ICD codes for inpatient and outpatient specialised healthcare when using the Swedish National Patient Register as a data source. Data on the American Society of Anaesthesiologists’ physical status classification (ASA-class) are presented descriptively; however, data on ASA were not used in the multivariable regression analysis as diabetes diagnosis itself is a component in the classification system.^23^ Demographic and baseline data to describe the T2DM population were obtained from the National Diabetes Register.

### Statistical analysis

The data were analysed using R V.4.2.0 (R Core Team 2022, Vienna, Austria). Descriptive data are presented using median and IQR. Outcome measures in terms of reoperation, adverse event and mortality were assessed unadjusted and adjusted for confounders, and in an explorative model also for mediators, listed in the previous section. Reoperation and mortality following OA-related primary hip or knee replacement were illustrated graphically using cumulative incidence (1 minus Kaplan‒Meier) curves with 95% CIs up to 12 and 10 years after primary hip and knee replacement. HRs were calculated using unadjusted and adjusted (multiple) Cox-regression models with 95% CI. Individuals were followed from the date of primary surgery until the first of: outcome event, death, migration out of Sweden or end of follow-up period (described in outcome paragraph) where they were censored in the analysis. Since reoperation, mortality and adverse events were rare (<10%) and the ORs themselves are not too large/small, unadjusted and adjusted OR could be used to estimate the relative risk (RR).^24 25^ When calculating adverse events using inpatient care data, the date of admission was used as a proxy for the date of adverse event diagnosis. Adverse events within 30/90 days were explored using logistic regression. All confounders and mediators were treated as categorical variables according to table 1 except for RX risk score that was treated as a continuous variable.

**Table 1:** Demographic and baseline characteristics in patients with primary hip replacement 2008‒2019 or primary knee replacement 2009‒2018 in Sweden, by diabetes status

Characteristics	Hip replacements		Knee replacements	
	NT2DM	T2DM	NT2DM	T2DM
Number of replacements	98 914	11 024	70 858	10 039
Confounding factors				
Age (median (IQR))	70.0(62.0, 76.0)	72.0(67.0, 78.0)	69.7(62.8, 75.9)	71.6(65.7, 76.7)
Age group (%)				
<50 years	3646 (3.7%)	53 (0.5%)	1620 (2.3%)	63 (0.6%)
50–59 years	13 878 (14.0%)	711 (6.4%)	10 129 (14.3%)	827 (8.2%)
60–69 years	31 634 (32.0%)	3155 (28.6%)	24 564 (34.7%)	3344 (33.3%)
70–79 years	35 752 (36.1%)	5127 (46.5%)	26 148 (36.9%)	4559 (45.4%)
≥80 years	14 004 (14.2%)	1978 (17.9%)	8397 (11.9%)	1246 (12.4%)
Female sex (%)	56 634 (57.3%)	5171 (46.9%)	40 978 (57.8%)	4923 (49.0%)
BMI (median (IQR))	26.6(24.2, 29.6)	29.07(26.2, 32.4)	28.1(25.4, 31.2)	30.5(27.5, 33.7)
BMI category (%)				
<18.5, underweight	724 (0.7%)	12 (0.1%)	149 (0.2%)	3 (0.0%)
18.5–24.9, normal weight	32 937 (33.3%)	1767 (16.0%)	14 969 (21.1%)	963 (9.6%)
25–29.9, overweight	42 780 (43.2%)	4553 (41.3%)	31 971 (45.1%)	3642 (36.3%)
30–34.9, class I obesity	17 627 (17.8%)	3325 (30.2%)	17 956 (25.3%)	3682 (36.7%)
35–39.9, class II obesity	4146 (4.2%)	1123 (10.2%)	4872 (6.9%)	1429 (14.2%)
≥ 40, class III obesity	700 (0.7%)	244 (2.2%)	941 (1.3%)	320 (3.2%)
Year of primary surgery (median (IQR))	2014(2011, 2017)	2015(2012, 2017)	2014(2011, 2017)	2014(2012, 2017)
2008/2009*–2010	19 067 (19.3%)	1821 (16.5%)	11 701 (16.5%)	1502 (15.0%)
2011–2013	22 459 (22.7%)	2516 (22.8%)	19 928 (28.1%)	2867 (28.6%)
2014–2016	25 500 (25.8%)	3034 (27.5%)	21 384 (30.2%)	2992 (29.8%)
2017–2018/2019†	31 888 (32.2%)	3653 (33.1%)	17 845 (25.2%)	2678 (26.7%)
Level of education (%)				
Primary and lower secondary education	28 230 (28.5%)	4299 (39.0%)	21 843 (30.8%)	3957 (39.4%)
Upper secondary education	41 860 (42.3%)	4646 (42.1%)	31 730 (44.8%)	4394 (43.8%)
Postsecondary education	27 782 (28.1%)	2036 (18.5%)	16 796 (23.7%)	1644 (16.4%)
Postgraduate education	1042 (1.1%)	43 (0.4%)	489 (0.7%)	44 (0.4%)
Index adjusted individual disposable income (100 SEK), median (IQR)‡	2185(1627, 3266)	1892(1525, 2692)	2148(1616, 3128)	1908(1523, 2652)
0–25% percentile	24 150 (24.4%)	3448 (31.3%)	17 210 (24.3%)	3024 (30.1%)
25–50% percentile	24 138 (24.4%)	3332 (30.2%)	17 241 (24.3%)	2976 (29.6%)
50–75% percentile	25 056 (25.3%)	2413 (21.9%)	17 964 (25.4%)	2263 (22.5%)
75–100% percentile	25 570 (25.9%)	1831 (16.6%)	18 443 (26.0%)	1776 (17.7%)
Modified RX risk score, median (IQR))	2(4)	3(4)	2(4)	3(4)
Explorative mediators				
Right-side surgery (%)	56 216 (56.8%)	6291 (57.1%)	37 879 (53.5%)	5192 (51.7%)
Incision (%)				
Anterior	45 063 (45.6%)	4908 (44.5%)	NA	NA
Posterior	53 098 (53.7%)	6041 (54.8%)	NA	NA
Other	753 (0.8%)	75 (0.7%)	NA	NA
Fixation (%)				
Cemented	61 522 (62.2%)	7956 (72.2%)	67 116 (94.7%)	9632 (95.9%)
Cementless	22 000 (22.2%)	1563 (14.2%)	3543 (5.0%)	391 (3.9%)
Hybrid	3337 (3.4%)	397 (3.6%)	199 (0.3%)	16 (0.2%)
Reverse hybrid	12 055 (12.2%)	1108 (10.1%)	NA	NA
ASA classification (%)§				
I	24 549 (25.0%)	195 (1.8%)	14 720 (20.8%)	138 (1.4%)
II	59 521 (60.7%)	6534 (59.8%)	46 274 (65.4%)	6323 (63.1%)
III	13 757 (14.0%)	4089 (37.4%)	9667 (13.7%)	3495 (34.9%)
≥IV	295 (0.3%)	112 (1.0%)	101 (0.1%)	61 (0.6%)

* Primary hip replacement 2008 or primary knee replacement 2009.

† Primary hip replacement 2019 or primary knee replacement 2018.

‡ Hip replacements: 0–25% percentile<1616 SEK; 25–50% percentile>=1616 SEK and < 2149 SEK; 50–75% percentile>=2149 SEK and <3215 SEK; and 75–100% percentile>=3215 SEK. Knee replacements: 0–25% percentile<1602 SEK; 25–50% percentile>=1602 SEK and < 2110 SEK; 50–75% percentile>=2110 SEK and < 3070 SEK; and 75–100% percentile>=3070 SEK.

§ ASA classification is missing in 0.8% and 0.9% of the registrations for NT2DM and T2DM, respectively. All other data are complete.

ASA, American Society of Anesthesiologists (classification of physical status); BMI, body-mass index; NT2DM, no diabetes population; RX risk score, risk score calculated as described in methods according to Pratt *et al*22; SEK, Swedish krona; T2DM, type 2 diabetes mellitus.

### Missing data

The completeness of primary total hip or knee replacements in the Swedish Arthroplasty Register 2022 was 97.9% and 97.5% when compared with the National Patient Register, which is mandatory by law to register.^26^ Despite the obligation to register, it is known that a small number of registrations are missing in the National Patient Register.^26^ The National Diabetes Register had an estimated completeness for adults aged 50‒79 years of 85% in 2022 when compared with individuals with filled prescriptions for diabetes drugs from the Prescribed Drug Register.^27^ Data from other sources are mandatory by law to record.

### Patient and public involvement

None.

## Results

109 938 hip replacements (98 914 without and 11 024 with T2DM) and 80 897 knee replacements (70 858 without and 10 039 with T2DM) cases were included in the study. The median follow-up time for patients with NT2DM and T2DM was 4.35 (IQR 1.94, 7.34) and 3.91 (IQR 1.72, 6.71) years for hip replacements and 4.04 (IQR 1.78, 6.63) and 3.81 (IQR 1.64, 6.23) years for knee replacements, respectively. Demographics and baseline characteristics are presented in table 1 and T2DM-specific characteristics are presented in table 2. Results presented as frequency in outcome are shown in table 3. Comorbidity/RX risk score profiles for the study groups are available in online supplemental figures 1 and 2.

**Table 2:** Diabetes-related demographic and baseline characteristics in T2DM patients with primary hip and knee replacement in Sweden in 2008‒2019 and 2009‒2018, respectively

Baseline characteristics	Hip replacements		Knee replacements	
		% missing		% missing
Individuals with diabetes diagnosis (n)	11 024	0%	10 039	0%
Days between initial enrolment in NDR and primary joint replacement (median (IQR))	140(303, 55)	0%	131(285, 52)	0%
HbA1c mmol/mol, within 18 months before primary joint replacement (median (IQR))	49.0(44.0, 56.0)	14%	50.0(44.0, 57.0)	13%
LDL-cholesterol mmol/l, within 6 months before primary joint replacement (median (IQR))	2.0(1.0, 3.0)	63%	2.0(1.0, 3.0)	62%
Systolic blood pressure, mm Hg, within 6 months before primary joint replacement (median (IQR))	135.0(127.0, 145.0)	46%	135.0(127.5, 144.0)	44%
Diastolic blood pressure, mm Hg, within 6 months before primary joint replacement (median (IQR))	76.0(70.0, 80.0)	46%	78.0(70.0, 80.0)	44%
Retinopathy, within 18 months before primary joint replacement (n (%))		34%		33%
No	5483 (75.5%)		4938 (73.7%)	
Yes	1780 (24.5%)		1760 (26.3%)	
Albuminuria, within 18 months before primary joint replacement (n (%))		33%		31%
No	5630 (76.0%)		5243 (76.1%)	
Normalised value	122 (1.6%)		105 (1.5%)	
Microalbuminuria	1250 (16.9%)		1183 (17.2%)	
Macroalbuminuria	409 (5.5%)		360 (5.2%)	
Diabetes treatment within 18 months before primary joint replacement (n (%))		13%		12%
Diet only	2275 (23.6%)		1990 (22.5%)	
Tablet (not GLP-1)	4839 (50.3%)		4323 (48.9%)	
Insulin	801 (8.3%)		744 (8.4%)	
Tablet (not GLP-1) and insulin	1477 (15.3%)		1523 (17.2%)	
GLP-1 analogue (injection/tablet)	16 (0.2%)		19 (0.2%)	
GLP-1 + tablet	102 (1.1%)		111 (1.3%)	
GLP-1 + insulin	32 (0.3%)		33 (0.4%)	
GLP-1 + tablet and insulin	85 (0.9%)		90 (1.0%)	
Lipid lowering medication within 18 months before primary joint replacement (n (%))		14%		14%
No	3294 (34.9%)		3097 (35.8%)	
Yes	6135 (65.1%)		5563 (64.2%)	
Blood pressure medication within 18 months before primary joint replacement (n (%))		14%		13%
No	1407 (14.8%)		1430 (16.4%)	
Yes	8076 (85.2%)		7293 (83.6%)	

GLP-1, glucagon-like peptide-1; HbA1c, hemoglobin A1c; LDL, low density lipoprotein; mm Hg, millimetre of mercury; n, number of; NDR, national diabetes register; T2DM, type 2 diabetes mellitus.

**Table 3:** Number and proportion of reoperations within 90 days, adverse events within 30 and 90 days and mortality within 90 days after primary hip and knee replacement in Sweden in 2008‒2019 and 2009‒2018, respectively

Outcome	Hip replacement		Knee replacement	
	NT2DM	T2DM	NT2DM	T2DM
Replacements at baseline, n	98 914	11 024	70 858	10 039
Type of reoperation during the study period, n (%)				
Other open surgical reoperation procedures	631 (21.5%)	98 (24.5%)	NA	NA
Revision	2300 (78.5%)	302 (75.5%)	1810 (100.0%)	287 (100.0%)
Reoperation within 90 days, n (%)	1087 (1.1%)	210 (1.9%)	431 (0.6%)	90 (0.9%)
Adverse events within 30 days, n (%)	3556 (3.6%)	528 (4.8%)	2210 (3.1%)	384 (3.8%)
Adverse events within 90 days, n (%)	4615 (4.7%)	715 (6.5%)	3329 (4.7%)	550 (5.5%)
Mortality within 90 days, n (%)	226 (0.2%)	48 (0.4%)	128 (0.2%)	29 (0.3%)

Adverse event is based on a selection of ICD-10 and NOMESCO codes specifically relevant following hip and knee replacement originally published by The Swedish Arthroplasty Register^21^; see Patients and Methods—Outcome measures for more information.

ICD-10, International Classification of Diseases version 10; n, number of; NA, Not applicable (other open surgical reoperation procedures were not obtained regarding knee replacements); NOMESCO, Nordic Medico-Statistical Committee; NT2DM, no diabetes population; T2DM, type 2 diabetes mellitus.

### Unadjusted analysis

**Figure 2:**
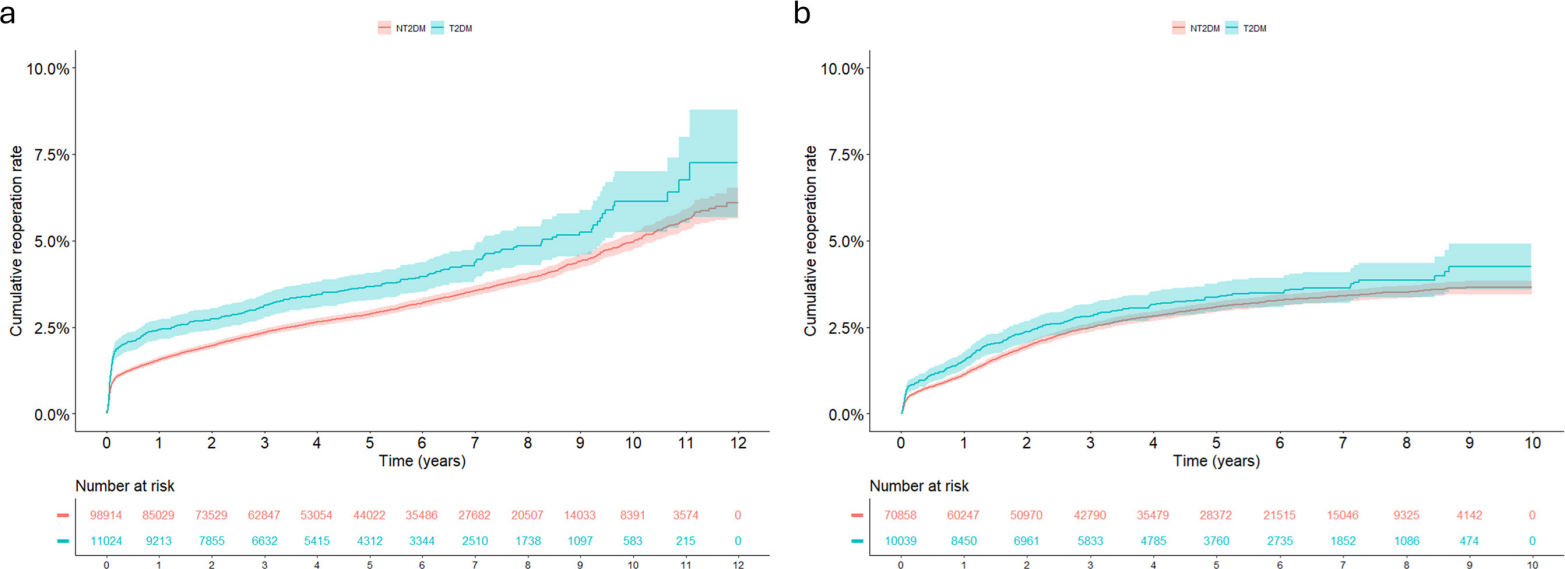
Unadjusted cumulative reoperation rate (1 minus Kaplan‒Meier) after primary hip and knee replacement in Sweden 2008‒2019 and 2009‒2018, respectively. NT2DM, no type 2 diabetes; T2DM, type 2 diabetes mellitus. (**a**) Reoperation after hip replacement. (**b**) Reoperation after knee replacement.

In the crude Kaplan‒Meier (cumulative reoperation rate (1 minus Kaplan‒Meier)) curves, individuals with diabetes showed increased risk of reoperation up to 10 years after hip replacement compared with individuals with no T2DM, while after knee replacement, an increased risk was limited to the first 2 years after primary surgery (figure 2). Unadjusted Cox-regression analysis indicated a T2DM-associated risk of reoperation, HR 1.30 (95% CI 1.18 to 1.45) for hip replacements and HR 1.15 (95% CI 1.01 to 1.30) for knee replacements. For cumulative incidence of mortality, the Kaplan‒Meier curves suggested a T2DM-related increased mortality risk after both hip and knee replacement (online supplemental figure 3). Using unadjusted Cox regression, T2DM was associated with mortality for hip (HR 1.84, 95% CI 1.76 to 1.9) and knee (HR 1.66, 95% CI 1.58 to 1.74) replacement. Unadjusted logistic regression analysis suggested that T2DM was associated with an increase in OR for reoperation, adverse events and 90-day mortality after hip and knee replacement (table 4).

**Table 4:** Unadjusted and adjusted odds ratios of reoperations within 90 days, adverse events within 30 and 90 days and mortality within 90 days in patients with or without T2DM after primary hip and knee replacement, respectively

Outcome	Hip replacement				Knee replacement			
	OR	95% CI	aOR	95% aCI	OR	95% CI	aOR	95% aCI
Reoperation within 90 days	1.75	1.50 to 2.02	1.23	1.05 to 1.43	1.48	1.17 to 1.85	1.08	0.85 to 1.36
Adverse events within 30 days	1.35	1.23 to 1.48	1.01	0.91 to 1.11	1.24	1.10 to 1.38	1.05	0.93 to 1.17
Adverse events within 90 days	1.42	1.31 to 1.54	1.07	0.98 to 1.16	1.18	1.07 to 1.29	1.08	0.98 to 1.19
90 days mortality	1.91	1.38 to 2.58	1.42	1.01 to 1.95	1.60	1.05 to 2.36	1.29	0.84 to 1.94

Adverse event is based on a selection of ICD-10 and NOMESCO codes specifically relevant following hip and knee replacement originally published by The Swedish Arthroplasty Register^21^; see Patients and Methods—Outcome measures for more information.

aOR/aCI, OR/CI adjusted for BMI, sex, age, level of education, income level, year of surgery and comorbidities; ICD-10, International Classification of Diseases version 10; NOMESCO, Nordic Medico-Statistical Committee; T2DM, type 2 diabetes mellitus.

### Adjusted analysis

After adjustment for confounders, Cox-regression analysis did not show a statistically significant increased risk associated with T2DM for reoperation after hip replacement or knee replacement (HR 1.10, 95% CI 0.99 to 1.23 and HR 1.09, 95% CI 0.96 to 1.24, respectively, table 5). However, T2DM was associated with an increase in mortality after hip replacement (HR 1.40, 95% CI 1.34 to 1.47) and after knee replacement (HR 1.38, 95% CI 1.31 to 1.45, table 6). The confounding factors age, BMI, sex and comorbidities were associated with the risk of reoperation and mortality, while level of education, income level and year of primary surgery were only associated with mortality and not risk of reoperation (tables5  6). For the risk of early (within 90 days) reoperation of the hip and mortality, a T2DM-associated increase was suggested after adjustment for confounders, OR 1.23 (95% CI 1.05 to 1.43) and 1.42 (95% CI 1.01 to 1.95), respectively (table 4). Corresponding adjusted ORs for adverse events within 30 and 90 days after hip replacement as well as reoperation within 90 days, and adverse events within 30 and 90 days and 90-day mortality after knee replacement showed no T2DM-associated increased odds (table 4). When explorative mediators were included in the models, the results did not differ notably.

**Table 5:** Adjusted Cox-regression analysis of the risk of hip and knee reoperation in patient with or without T2DM after primary hip and knee replacement, respectively

Baseline characteristics	Hip replacement		Knee replacement	
	HR	95% CI	HR	95% CI
T2DM	1.10	0.99 to 1.23	1.09	0.96 to 1.24
Age groups				
Less than 50 years	Ref		Ref	
50–59 years	0.90	0.75 to 1.08	0.56	0.45 to 0.69
60–69 years	0.77	0.65 to 0.92	0.37	0.31 to 0.46
70–79 years	0.74	0.62 to 0.89	0.34	0.28 to 0.42
≥80 years	0.86	0.71 to 1.05	0.30	0.24 to 0.39
BMI category				
18.5–24.9, normal weight	Ref		Ref	
<18.5, underweight	1.21	0.78 to 1.89	0.33	0.05 to 2.38
25–29.9, overweight	1.12	1.03 to 1.22	1.11	0.98 to 1.26
30–34.9, class I obesity	1.42	1.29 to 1.57	1.33	1.16 to 1.52
35–39.9, class II obesity	1.82	1.58 to 2.11	1.23	1.02 to 1.48
≥ 40, class III obesity	2.10	1.60 to 2.76	1.32	0.96 to 1.81
Sex				
Female	Ref		Ref	
Male	1.46	1.36 to 1.57	1.20	1.09 to 1.32
Level of education				
Primary and lower secondary education	Ref		Ref	
Upper secondary education	1.08	0.99 to 1.17	0.97	0.88 to 1.07
Postsecondary education	0.95	0.86 to 1.05	0.88	0.78 to 1.01
Postgraduate education	0.78	0.52 to 1.18	0.99	0.56 to 1.76
Index adjusted individual disposable income (100 SEK)				
0–25 percentile	Ref		Ref	
25–50 percentile	1.03	0.93 to 1.14	0.96	0.84 to 1.08
50–75 percentile	0.98	0.88 to 1.09	0.96	0.85 to 1.10
75–100 percentile	1.04	0.92 to 1.16	0.88	0.76 to 1.01
Year of primary surgery				
2008/2009*‒2010	Ref		Ref	
2011‒2013	0.92	0.83 to 1.01	1.12	0.99 to 1.26
2014‒2016	0.89	0.81 to 0.99	1.12	0.99 to 1.27
2017‒2018/2019†	0.96	0.86 to 1.07	1.04	0.88 to 1.24
Comorbidities—Rx risk score	1.06	1.05 to 1.07	1.05	1.04 to 1.07

* 2008 for hip replacements and 2009 for knee replacements.

† 2018 for knee replacements and 2019 for hip replacements.

BMI, body-mass index; Ref, reference; RX risk score, risk score calculated as described in methods according to Pratt *et al*22; SEK, Swedish krona; T2DM, type 2 diabetes mellitus.

**Table 6:** Adjusted Cox-regression analysis of the risk of death after hip and knee reoperation in patient with or without T2DM after primary hip and knee replacement, respectively

Baseline characteristics	Hip replacement		Knee replacement	
	HR	95% CI	HR	95% CI
T2DM	1.40	1.34 to 1.47	1.38	1.31 to 1.45
Age				
Less than 50 years	Ref		Ref	
50–59 years	2.48	1.83 to 3.36	1.51	1.08 to 2.11
60–69 years	4.91	3.67 to 6.58	2.90	2.10 to 3.99
70–79 years	11.2	8.35 to 14.9	6.56	4.76 to 9.03
≥80 years	28.1	21.0 to 37.6	16.7	12.1 to 23.0
BMI				
18.5–24.9, normal weight	Ref		Ref	
<18.5, underweight	1.55	1.34 to 1.80	2.09	1.56 to 2.81
25–29.9, overweight	0.84	0.81 to 0.87	0.85	0.81 to 0.89
30–34.9, class I obesity	0.87	0.83 to 0.92	0.87	0.82 to 0.92
35–39.9, class II obesity	1.08	0.99 to 1.17	1.08	0.99 to 1.17
≥ 40, class III obesity	1.35	1.15 to 1.59	1.25	1.08 to 1.45
Sex				
Female	Ref		Ref	
Male	1.66	1.61 to 1.72	1.71	1.64 to 1.78
Level of education				
Primary and lower secondary education	Ref		Ref	
Upper secondary education	0.94	0.91 to 0.97	0.92	0.88 to 0.96
Postsecondary education	0.81	0.77 to 0.85	0.86	0.81 to 0.91
Postgraduate education	0.73	0.59 to 0.90	0.85	0.65 to 1.11
Index adjusted individual disposable income (100 SEK)				
0–25 percentile	Ref		Ref	
25–50 percentile	1.01	0.97 to 1.05	0.97	0.92 to 1.02
50–75 percentile	0.87	0.83 to 0.91	0.86	0.81 to 0.91
75–100 percentile	0.70	0.66 to 0.74	0.70	0.65 to 0.75
Year of primary surgery				
2008/2009*‒2010	Ref		Ref	
2011‒2013	0.88	0.85 to 0.92	0.87	0.83 to 0.91
2014‒2016	0.79	0.75 to 0.83	0.84	0.79 to 0.89
2017‒2018/2019†	0.68	0.63 to 0.74	0.68	0.61 to 0.75
Comorbidities—Rx risk score	1.09	1.09 to 1.10	1.09	1.08 to 1.09

22

* 2008 for hip replacements and 2009 for knee replacements.

† 2018 for knee replacements and 2019 for hip replacements.

BMI, body-mass index; Ref, reference; RX risk score, risk score calculated as described in methods according to Pratt *et al*22; SEK, Swedish krona; T2DM, type 2 diabetes mellitus.

## Discussion

This population-based observational study found that, in general, hip and knee replacements were considered safe for individuals with type 2 diabetes. When investigating events during the follow-up time using Cox regression, no associations were observed between T2DM and reoperation or between T2DM and adverse events after OA-related hip and knee replacement compared with individuals with NT2DM. Investigating short-term odds for reoperation (within 90 days) with a logistic regression model suggests an increase in T2DM-associated odds after hip replacement (23%) but not after knee replacement. A Danish study has previously published similar data with a type 2 diabetes-associated RR of 1.15 (95% CI 0.97 to 1.37) for all cause revision after total hip replacement during the study period (1996‒2005).^10^ Additionally, in the study, they found no short-term RR of revision for all causes within 2 years in total hip replacement (1.11 (95% CI 0.94 to 1.32)).^10^ In the Danish study, the data were not adjusted for BMI; however, hospital diagnosis of obesity was used as a confounder.^10^ Further, a study from Kaiser Permanente, USA, showed uncertain association between controlled or uncontrolled diabetes (HbA1c<7% and >7%) in terms of revision after total knee replacement (OR 1.32, 95% CI 0.99 to 1.76, and OR 1.03, 95% CI 0.68 to 1.54, respectively).^16^ The analysis was adjusted for BMI. A meta-analysis performed by Podmore *et al* showed that diabetes was associated with increased odds of revision after total hip or knee replacement combined, OR 1.28 (95% CI 1.02 to 1.59).^13^

T2DM was not shown to be associated with increased odds of adverse events within 30 and 90 days after hip or knee replacement using the Swedish Arthroplasty Register’s definition of adverse events. This may, however, not be fully comparable with definitions used in other studies. A limitation of this study is that adverse events were only captured through specialist and inpatient care records. It is likely that less severe events were managed in primary care, for which registry data were not available. A T2DM-associated increased risk of death by 40% and 38%, respectively, after hip and knee replacement was observed using a multivariable Cox-regression analysis. Logistic regression analysis investigating mortality within 90 days after hip replacement showed a 42% T2DM-associated increase in odds of death. For knee replacement, however, there was no clear increased odds of death within 90 days. It should be noted that short-term mortality is rare following hip and knee replacement, and the CIs were wide. Limitations in the study design (with no comparative study group not undergoing hip or knee replacement) make it impossible to distinguish whether the observed increased mortality was linked to hip or knee replacement surgery or to the diabetes diagnosis itself. Diabetes is known to be associated with higher mortality.^28 29^ It is reasonable to think that short-term mortality is more likely to be associated with the replacement surgery than long-term mortality. In this study, confounding factors shown to have an impact on mortality were age, BMI, sex, level of education, level of income, year of surgery and comorbidities as measured by the RX risk score. Likely, there are several factors not included in the study that may influence the outcome in terms of mortality, for example, smoking status, exercise status and comorbidities not covered by the RX risk score. Other studies investigating T2DM-associated mortality after hip or knee replacement often focus on in-hospital or short-term mortality. A Spanish study reported higher in-hospital mortality after total knee replacement in individuals with diabetes compared with matched controls (0.09% vs 0.02%, p=0.002).^15^ Corresponding data after hip replacement were (0.25% vs 0.19%, p=0.388).^15^ The control group was not matched on BMI. A study from the USA reports no diabetes-associated inpatient mortality after total hip and knee replacement, OR 1.0 (95% CI 0.8 to 1.3) and 0.9 (95% CI 0.7 to 1.2), respectively, also unadjusted for BMI.^12^ Further, a meta-analysis by Podmore *et al* showed a diabetes-associated increase in short-term mortality (maximum 3 months after surgery), OR 1.26 (95% CI 1.15 to 1.38), for total hip and knee replacement combined while they found no diabetes-associated long-term mortality (closest to 12 months after surgery), OR 0.97, 95% CI 0.82 to 1.13.^13^

One drawback with register studies is that the variables collected are predetermined and the possibility to add relevant information is limited. For instance, glycaemic control, measured by HbA1c, would have been a valuable covariate in this study; however, these data were not consistently available across the entire study cohort. Another limitation with this study that may influence the results is that joint replacement registrations with incomplete data were excluded in the data analysis (about 4% for hip and 2% for knee). Hip or knee replacements or T2DM diagnoses not registered in the national quality registers are, however, expected to be few, based on previously presented completeness. In the case of missing events and data, there is no reason to suspect differential missingness between the groups, implying minimal impact on the end results. The study is based on the data from a Swedish cohort and even though it is reasonable to assume that the results may have relevance to other countries, there might be national differences between countries, for example, regarding diet and exercise as well as healthcare practices that should be considered. Moreover, in this study, we have not included data describing whether the included patients have completed basic treatment for OA before surgery according to the international guidelines. It should also be noted that individuals included close to the end of the study period does not contribute with a complete follow-up time for reoperation and after hip replacement for adverse events, when restricting the follow-up to 30 or 90 days, as end of inclusion and end of follow-up were both 31 December 2019. Based on the length of the study period and included individuals, this will have minimal impact on the results. When interpreting the data, it is important to keep in mind that only those individuals with T2DM who have been selected as suitable to undergo hip replacement surgery were included in the study.

The strength of this study is that it is based on close to complete coverage in the Swedish population. Sweden has unique possibilities to link data from different sources using a national personal identity number. This, in combination with a legislation that supports collection and linkage of data for research, based on ethical approval supports the creation of unique datasets. Specific informed consent is not required from study participants as the national quality register system is based on information at the time of surgery and opt-out, thus enabling representative study cohorts. The years of the COVID-19 pandemic are not included in the study, making the results more relevant to ‘normal’ healthcare conditions. Further, in the study, analyses were adjusted for key confounding factors, such as age and BMI. Only a limited number of previous studies included adjustment for BMI. Another advantage was that the analysis of hip and knee replacements was performed in parallel using the same methodology, allowing for comparison between hip and knee replacement.

In summary, no T2DM-associated increase in risks of reoperation or adverse events after knee replacement was observed in this study. Similarly, there was no T2DM-associated increase in risk of early adverse events (within 30/90 days) after hip replacement; however, an increased risk of early reoperation (within 90 days) was seen. This was not confirmed using a Cox-regression analysis including events during a longer follow-up time. It is important to remember that individuals included in this study already were selected suitable for hip and knee surgery and it may be possible that the decision whether to operate or not was influenced by diabetes status. In this context, the study would appear to confirm that the decision to operate individuals with T2DM was in many cases the right decision. Further studies are needed to investigate potential positive impact on optimised T2DM control prior to and after hip and knee replacement.

In conclusion, joint replacement surgery, whether it is in the hip or knee, may, in general, be considered safe in terms of complications, such as reoperation, adverse events and mortality, in selected individuals both with and without T2DM.

## Supplementary material

10.1136/bmjopen-2024-096717 online supplemental file 1

## Data Availability

No data are available.

## References

[1] Sun H, Saeedi P, Karuranga S (2022). IDF Diabetes Atlas: Global, regional and country-level diabetes prevalence estimates for 2021 and projections for 2045. Diabetes Res Clin Pract.

[2] Steinmetz JD, Culbreth GT, Haile LM (2023). Global, regional, and national burden of osteoarthritis, 1990–2020 and projections to 2050: a systematic analysis for the Global Burden of Disease Study 2021. The Lancet Rheumatology.

[3] Centers for Disease Control and Prevention (CDC) (2008). Arthritis as a potential barrier to physical activity among adults with diabetes--United States, 2005 and 2007. MMWR Morb Mortal Wkly Rep.

[4] Piva SR, Susko AM, Khoja SS (2015). Links between osteoarthritis and diabetes: implications for management from a physical activity perspective. Clin Geriatr Med.

[5] Jönsson T, Eek F, Dell’Isola A (2019). The Better Management of Patients with Osteoarthritis Program: Outcomes after evidence-based education and exercise delivered nationwide in Sweden. PLoS ONE.

[6] Katz JN, Arant KR, Loeser RF (2021). Diagnosis and Treatment of Hip and Knee Osteoarthritis: A Review. JAMA.

[7] Puhto T, Puhto A-P, Vielma M (2019). Infection triples the cost of a primary joint arthroplasty. Infect Dis (Lond).

[8] Wildeman P, Rolfson O, Söderquist B (2021). What Are the Long-term Outcomes of Mortality, Quality of Life, and Hip Function after Prosthetic Joint Infection of the Hip? A 10-year Follow-up from Sweden. Clin Orthop Relat Res.

[9] Nemes S, Gordon M, Rogmark C (2014). Projections of total hip replacement in Sweden from 2013 to 2030. Acta Orthop.

[10] Pedersen AB, Mehnert F, Johnsen SP (2010). Risk of revision of a total hip replacement in patients with diabetes mellitus: a population-based follow up study. J Bone Joint Surg Br.

[11] Qin W, Huang X, Yang H (2020). The Influence of Diabetes Mellitus on Patients Undergoing Primary Total Lower Extremity Arthroplasty: A Systematic Review and Meta-Analysis. Biomed Res Int.

[12] Bolognesi MP, Marchant MH, Viens NA (2008). The impact of diabetes on perioperative patient outcomes after total hip and total knee arthroplasty in the United States. J Arthroplasty.

[13] Podmore B, Hutchings A, van der Meulen J (2018). Impact of comorbid conditions on outcomes of hip and knee replacement surgery: a systematic review and meta-analysis. BMJ Open.

[14] Jain NB, Guller U, Pietrobon R (2005). Comorbidities increase complication rates in patients having arthroplasty. Clin Orthop Relat Res.

[15] Martínez-Huedo MA, Jiménez-García R, Jiménez-Trujillo I (2017). Effect of Type 2 Diabetes on In-Hospital Postoperative Complications and Mortality After Primary Total Hip and Knee Arthroplasty. J Arthroplasty.

[16] Adams AL, Paxton EW, Wang JQ (2013). Surgical outcomes of total knee replacement according to diabetes status and glycemic control, 2001 to 2009. J Bone Joint Surg Am.

[17] Lenguerrand E, Beswick AD, Whitehouse MR (2018). Outcomes following hip and knee replacement in diabetic versus nondiabetic patients and well versus poorly controlled diabetic patients: a prospective cohort study. Acta Orthop.

[18] Dell’Isola A, Vinblad J, Lohmander S (2019). Understanding the role of diabetes in the osteoarthritis disease and treatment process: a study protocol for the Swedish Osteoarthritis and Diabetes (SOAD) cohort. BMJ Open.

[19] Bülow E, Nemes S, Rolfson O (2020). Are the First or the Second Hips of Staged Bilateral THAs More Similar to Unilateral Procedures? A Study from the Swedish Hip Arthroplasty Register. Clin Orthop Relat Res.

[20] Ludvigsson JF, Otterblad-Olausson P, Pettersson BU (2009). The Swedish personal identity number: possibilities and pitfalls in healthcare and medical research. Eur J Epidemiol.

[21] W-Dahl A, Kärrholm J, Rogmark C (2021). The swedish arthroplastry register annual report 2021.

[22] Pratt NL, Kerr M, Barratt JD (2018). The validity of the Rx-Risk Comorbidity Index using medicines mapped to the Anatomical Therapeutic Chemical (ATC) Classification System. BMJ Open.

[23] Mayhew D, Mendonca V, Murthy BVS (2019). A review of ASA physical status - historical perspectives and modern developments. Anaesthesia.

[24] Ranganathan P, Aggarwal R, Pramesh CS (2015). Common pitfalls in statistical analysis: Odds versus risk. Perspect Clin Res.

[25] Davies HTO, Crombie IK, Tavakoli M (1998). When can odds ratios mislead?. BMJ.

[26] W-Dahl A, Kärrholm J, Rogmark C (2023). Svenska ledprotesregistrets årsrapport 2023.

[27] Eeg-Olofsson K, Åkesson K, Thorén A (2023). Nationella diabetesregistret årsrapport 2022.

[28] Li S, Wang J, Zhang B (2019). Diabetes Mellitus and Cause-Specific Mortality: A Population-Based Study. Diabetes Metab J.

[29] Rawshani A, Rawshani A, Franzén S (2017). Mortality and Cardiovascular Disease in Type 1 and Type 2 Diabetes. N Engl J Med.

