# Supplementary material for: Association of type 2 diabetes with reoperation, adverse events and mortality after hip and knee replacement: a Swedish register-based study including 109 938 hip and 80 897 knee replacements
Source: BMJ Open. 2025 Sep 28;15(9):e096717. doi: 10.1136/bmjopen-2024-096717 (PMC12481289; doi:10.1136/bmjopen-2024-096717)
Supplement: online supplemental file 1 [file bmjopen-15-9-s001.docx]

## **Supplementary figures**

**Supplementary Figure 1.** RX risk score (as defined by Pratt *et al*^1^) distribution between study groups at baseline (one year prior to surgery) for patients undergoing primary hip and knee replacement in Sweden 2008‒2019 and 2009‒2018, respectively. Diabetes is not included as it is defined as exposure parameter in the study. NT2DM = no diabetes population, T2DM = type 2 diabetes mellitus.


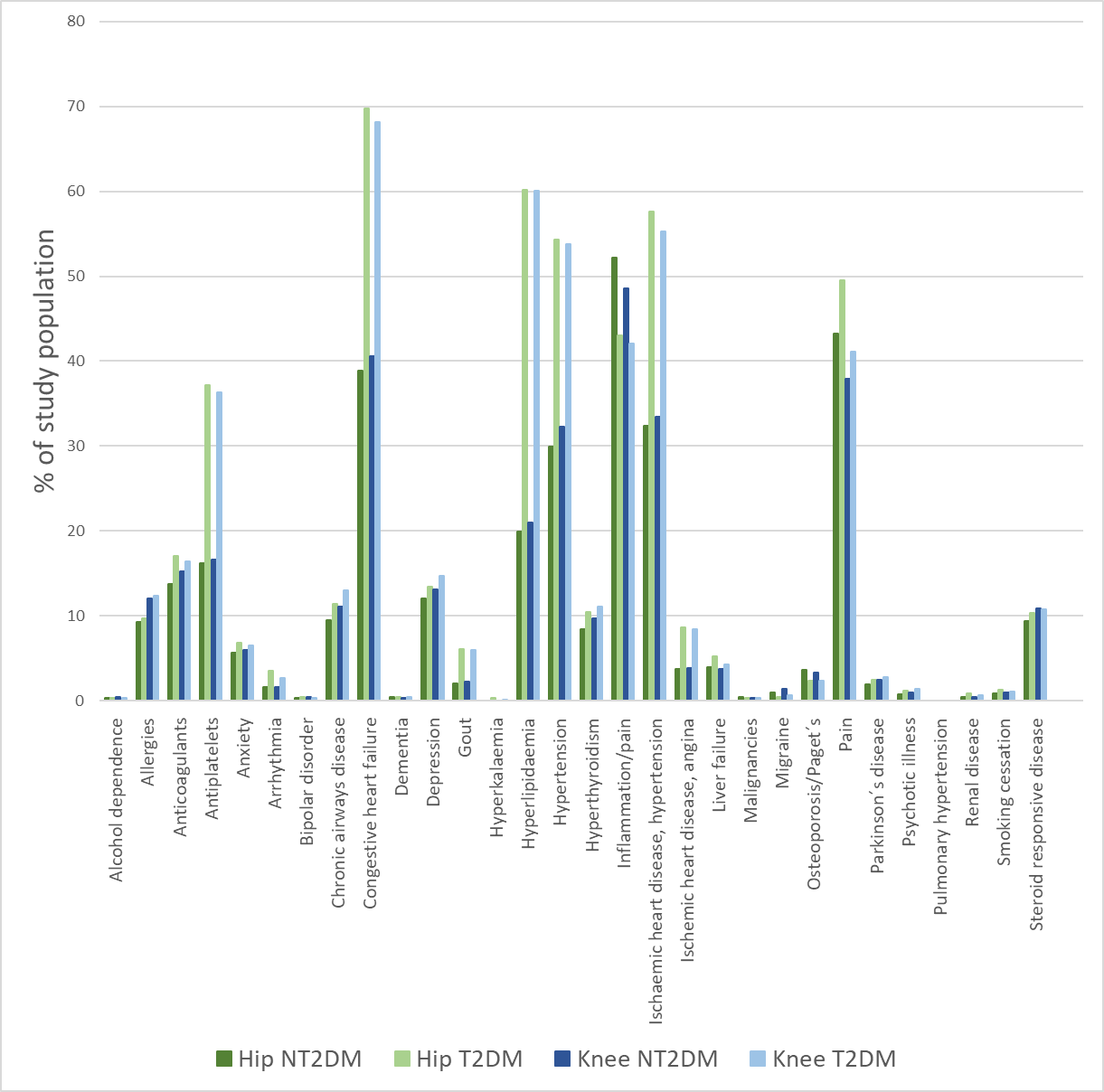


**Supplementary Figure 2.** Distribution of RX risk score as defined by Pratt *et al* ^1^ between diagnosis and study groups at baseline (one year prior to surgery) for patients undergoing primary hip and knee replacement in Sweden 2008‒2019 and 2009‒2018, respectively. Benign prostatic hyperplasia, epilepsy, glaucoma, gastroesophageal reflux disease, hepatitis B, hepatitis C, HIV, hypothyroidism, irritable bowel syndrome, incontinence, malnutrition, pancreatic insufficiency, psoriasis, transplant and tuberculosis are not presented in the graph as they do not contribute to the total score (weigh 0). Diabetes is not included as it is defined as exposure parameter in the study. NT2DM = no diabetes population, T2DM = type 2 diabetes mellitus.

**3a)**

**
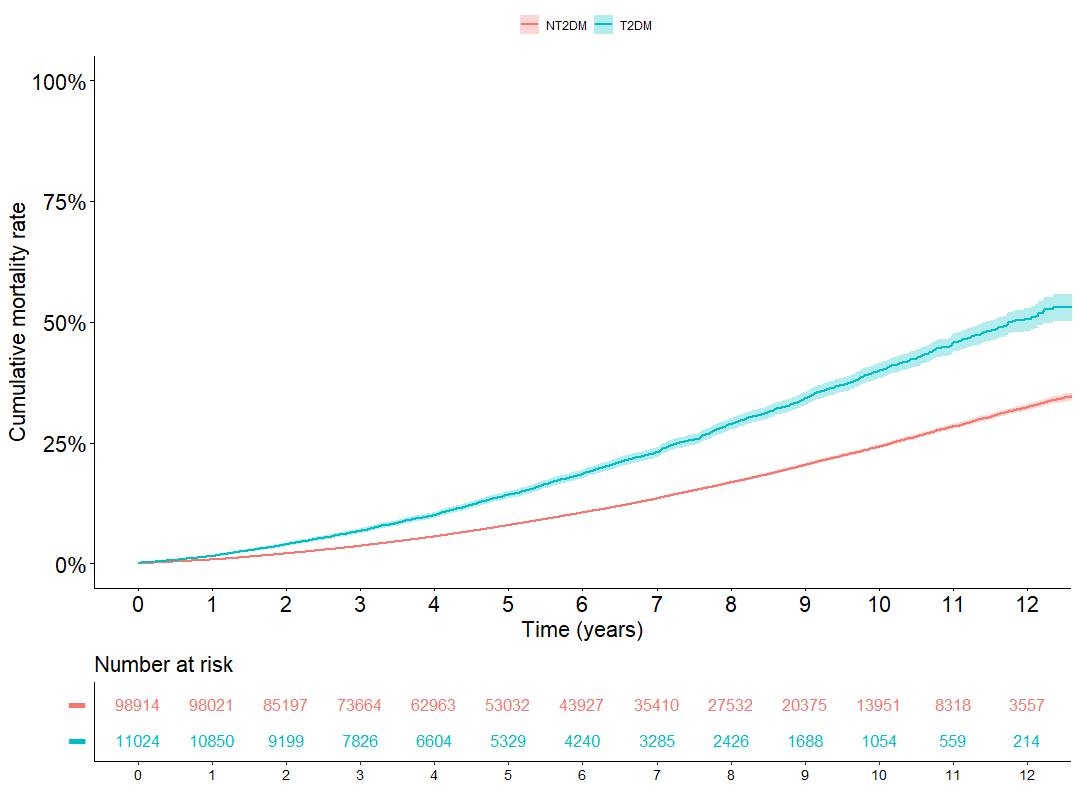
**

**3b)**

**
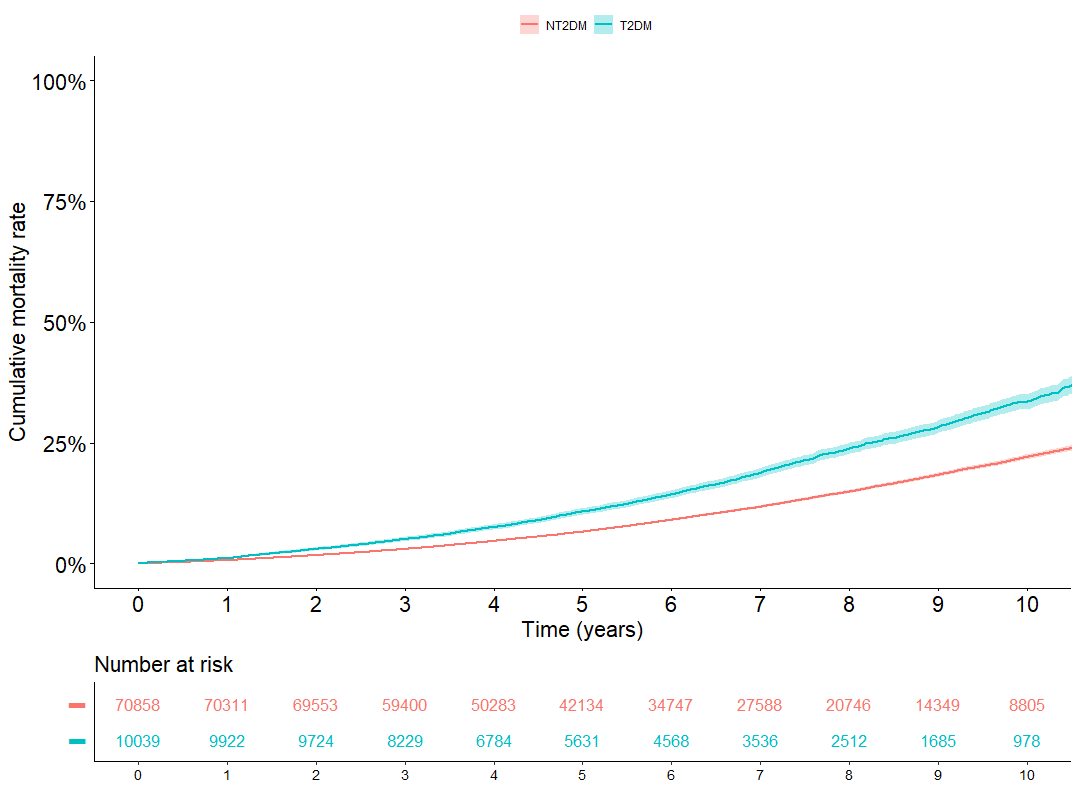
**

**Supplementary Figure 3.** Unadjusted cumulative mortality rate (1 minus Kaplan‒Meier) after primary hip and knee replacement in Sweden 2008‒2019 and 2009‒2018, respectively. NT2DM = no type 2 diabetes, T2DM = type 2 diabetes mellitus. a) Mortality after hip replacement. b) Mortality after knee replacement.

1. Pratt, N.L.*, et al.* The validity of the Rx-Risk Comorbidity Index using medicines mapped to the Anatomical Therapeutic Chemical (ATC) Classification System. *BMJ open* **8**, e021122 (2018).
