# Supplementary material for: Association of type 2 diabetes with reoperation, adverse events and mortality after hip and knee replacement: a Swedish register-based study including 109 938 hip and 80 897 knee replacements
Source: BMJ Open. 2025 Sep 28;15(9):e096717. doi: 10.1136/bmjopen-2024-096717 (PMC12481289; doi:10.1136/bmjopen-2024-096717)
Supplement: Supplementary file 2 [file bmjopen-2024-096717.reviewer_comments.pdf]

PEER REVIEW HISTORY

BMJ Open publishes all reviews undertaken for accepted manuscripts. Reviewers are asked to complete a checklist review form and are provided with free text boxes to elaborate on their assessment. These free text comments are reproduced below.

ARTICLE DETAILS

Title (Provisional)

Association of type 2 diabetes with reoperation, adverse events and mortality after hip and knee replacement: a Swedish register-based study including 109,938 hip and 80,897 knee replacements

Authors

Vinblad, Johanna; Bülow, Erik; Nyberg, Fredrik; Eeg-Olofsson, Katarina; W-Dahl, A; Limbäck, Gunilla; Englund, Martin; Abbott, Allan; Dell'Isola, Andrea; Rolfson, Ola

---

VERSION 1 - REVIEW

Reviewer **1**

Name **Babhulkar, Sushrut**

Affiliation **Sushrut Hospital, Orthopedics**

Date **29-Dec-2024**

COI **None**

Well written. Clear aims and objectives. This research will help many make a good clinical decision.

Reviewer **2**

Name **Agrawal, Yuvraj**

Affiliation **Royal Orthopaedic Hospital NHS Foundation Trust, Lower Limb Arthroplasty Unit**

Date **06-Jan-2025**

COI **None**

The paper is well written and provides further clarity on an important topic. All exclusions are accounted for. Patients with inaccurate data, missing data and even cross-checked for anti-hypoglycaemics was commendable.

Reviewer **3**

Name **Guo, Junfei**

Affiliation **Xi'an Jiaotong University, Department of Orthopaedic, Joint Surgery Surgery**

Date **23-Apr-2025**

COI **None**

This study investigates the association between T2DM and postoperative outcomes following hip and knee replacements using Swedish national registries. While the topic is clinically relevant and the large sample size (nearly 200,000 cases) is a strength, significant methodological and interpretative limitations undermine the validity and generalizability of the findings. Below, I outline specific major concerns that warrant rejection.

Major Concerns

1. The study adjusts for several confounders (e.g., age, BMI, comorbidities) but fails to account for critical factors influencing surgical outcomes in T2DM patients: HbA1c levels, a key determinant of diabetes severity and perioperative risk, are mentioned in Table 2 but not incorporated into adjusted analyses. Poor glycemic control is strongly associated with complications (e.g., infection, delayed healing), yet this variable is omitted.

2. Lifestyle Factors such as smoking, physical activity, and dietary habits—factors linked to both T2DM progression and surgical recovery—are not addressed.

3. The study period for hip replacements (2008–2019) and knee replacements (2009–2018) overlaps with evolving surgical techniques and diabetes management practices. Temporal trends (e.g., improved glycemic control protocols) are not addressed, potentially diluting associations.

4. Excluding bilateral procedures (45,558 hips and 38,248 knees) limits generalizability, as these patients may differ systematically (e.g., higher comorbidity burden).

5. The reliance on inpatient ICD-10/NOMESCO codes likely underestimates complications (e.g., outpatient infections, minor thromboembolic events). This misclassification biases results toward the null, particularly for T2DM patients, who may experience higher rates of subacute complications.

6. For knees, reoperation is restricted to revisions (implant exchange/removal), while hips include other open procedures. Heterogeneity in outcome definitions complicates comparisons between joints and with prior literature.

7. Mortality is a competing risk for reoperation. The use of Kaplan-Meier and Cox regression without competing risk analysis (e.g., Fine-Gray models) overestimates reoperation rates, particularly in T2DM patients with higher mortality.

8. The nonsignificant association between T2DM and knee reoperation (adjusted HR 1.09, 95% CI 0.96–1.24) may reflect inadequate power rather than a true null effect. A post-hoc

power calculation is needed to confirm whether the sample size was sufficient for subgroup analyses.

**Reviewer**	**4**
**Name**	**Dack, Kyle**
**Affiliation**	**University of Bristol, MRC Integrative Epidemiology Unit**
**Date**	**29-Jun-2025**
**COI**	**None**

Statistical review summary:

This is very well written, and clearly reports the methodology and results. The data is high-quality with an excellent sample size, and the authors have used reliably modelling methods which minimize the risk of bias. I have only a few minor suggestions to improve the clarity, but regarding the statistical analysis I have no concerns.

Abstract:

Clear – no suggestions.

Introduction:

Line 102: A reference is made to inconsistency in prior literature but I don't think the previous paragraph clearly showed what this inconsistency is. Is it that Lenguerrand et al did not find an association with T2DM? If so, I suggest directly reporting this because the current summary only implies it.

Methods:

Line 115. Is it possible to report a summary of what data was taken from each database? Even if perhaps just a summary table in the supplementary materials, this could be useful for readers wanting to know more about each data source.

Figure 1. Very clear and helpful but I suggest increasing resolution if possible because when zoomed the text is pixelated.

What was the rationale for the use of different end dates for risk of reoperation, mortality, and adverse events? If it was due to differences in data availability I suggest stating this for clarity.

Line 174. The authors stated that duration of disease diagnosis was not available. I wonder if this could be a potential mediator which future studies could explore? E.g. if there are different associations between those with long term diabetes vs those recently diagnosed. If this is a valid idea I suggest mentioning in the discussion?

Line 205. It is not clear from this whether the all models included mediators, or whether models with and without mediators were generated.

Results

IQR is reported as ICQ in places, one should be used for consistency.

Results are really clear and well reported, I have almost no suggestions. My only feedback is that this line: "The inclusion of the explorative mediators together with the confounding showed similar results (data not shown)." is jarring because it references results being showed, but then data not shown. I suggest either changing it to the negative "When explorative mediators were included in the models, the results did not differ notably", or even better to add those results as supplementary tables which can be referenced.

VERSION 1 - AUTHOR RESPONSE

Reviewer: 1
Dr. Sushrut Babhulkar, Sushrut Hospital

Comments to the Author:

- Well written. Clear aims and objectives. This research will help many make a good clinical decision.
    - Authors response: Thank you for your kind feedback. We're glad the study's aims came through clearly and that you see its potential to support clinical decision-making. We appreciate your encouraging words.

Reviewer: 2
Dr. Yuvraj Agrawal, Royal Orthopaedic Hospital NHS Foundation Trust

Comments to the Author:

- The paper is well written and provides further clarity on an important topic. All exclusions are accounted for. Patients with inaccurate data, missing data and even cross-checked for anti-hypoglycaemics was commendable.
    - Authors response: Thank you for your positive feedback. We're glad the clarity of the paper and our thorough approach to data validation were appreciated.

Reviewer: 3
Dr. Junfei Guo, Xi'an Jiaotong University

Comments to the Author:

- This study investigates the association between T2DM and postoperative outcomes following hip and knee replacements using Swedish national registries. While the topic is clinically relevant and the large sample size (nearly 200,000 cases) is a strength, significant methodological and interpretative limitations undermine the validity and generalizability of the findings. Below, I outline specific major concerns that warrant rejection.
    - Authors response: Thank you for your detailed review and for acknowledging the clinical relevance and sample size of our study. We appreciate your critical insights and take your concerns seriously. We will carefully address each methodological and interpretative point raised to strengthen the validity and generalizability of our findings.

Major Concerns

1. The study adjusts for several confounders (e.g., age, BMI, comorbidities) but fails to account for critical factors influencing surgical outcomes in T2DM patients: HbA1c levels, a key determinant of diabetes severity and perioperative risk, are mentioned in Table 2 but not incorporated into adjusted analyses. Poor glycemic control is strongly associated with complications (e.g., infection, delayed healing), yet this variable is omitted.
    - Authors response: Thank you for highlighting the importance of glycemic control in surgical outcomes. We agree that HbA1c is a clinically relevant variable. However, as HbA1c data were not available for the non-diabetic group, we were unable to include this variable in the adjusted analyses. Comment added in discussion section "For instance, glycaemic control, measured by HbA1c, would have been a valuable covariate in this study; however, these data were not consistently available across the entire study cohort." (row 320-322).

2. Lifestyle Factors such as smoking, physical activity, and dietary habits—factors linked to both T2DM progression and surgical recovery—are not addressed.
    - Authors response: Thank you for pointing out the relevance of lifestyle factors such as smoking, physical activity, and dietary habits, which are indeed associated with both the progression of type 2 diabetes and surgical recovery. Unfortunately, we did not have access to complete lifestyle data for the entire cohort. Dietary information was available only for participants with diabetes, limiting its use in broader analyses. While we did have some data on

smoking, it was incomplete — characterized by a high level of missingness and restricted to a limited number of years. To avoid introducing bias, we chose not to include smoking in our analysis.

3. The study period for hip replacements (2008–2019) and knee replacements (2009–2018) overlaps with evolving surgical techniques and diabetes management practices. Temporal trends (e.g., improved glycemic control protocols) are not addressed, potentially diluting associations.

- Authors response: Thank you for raising this important point regarding temporal trends in surgical techniques and diabetes management. We acknowledge that advancements over time could influence outcomes. To account for these potential changes, we included the year of surgery as a covariate in our analysis. This approach helps mitigate the impact of evolving practices on the observed associations.

4. Excluding bilateral procedures (45,558 hips and 38,248 knees) limits generalizability, as these patients may differ systematically (e.g., higher comorbidity burden).

- Authors response: Thank you for your comment. We excluded bilateral procedures to avoid introducing bias, as including both sides for a single patient would result in duplicate entries and violate the assumption of independent observations. The decision to include only the second replacement was based on conclusions drawn by Bülow et al (reference 19 in the manuscript).

5. The reliance on inpatient ICD-10/NOMESCO codes likely underestimates complications (e.g., outpatient infections, minor thromboembolic events). This misclassification biases results toward the null, particularly for T2DM patients, who may experience higher rates of subacute complications.

- Authors response: Thank you for this important observation. We acknowledge that relying on inpatient and specialist care ICD-10 and NOMESCO codes may lead to underestimation of certain complications, particularly those managed in outpatient settings. This is a limitation of the available data. However, we believe that the most severe adverse events—those most likely to impact clinical outcomes—were captured reliably through inpatient records. We have included this as a limitation in the discussion where limitations are discussed to ensure transparency regarding data constraints (row 291-294).

6. For knees, reoperation is restricted to revisions (implant exchange/removal), while hips include other open procedures. Heterogeneity in outcome definitions complicates comparisons between joints and with prior literature.

- Authors response: Thank you for your comment. For knees, reoperations are restricted to revisions involving implant exchange or removal, whereas for hips, the definition includes other open procedures. This heterogeneity in outcome definitions complicates direct comparisons between joints and with prior literature. The discrepancy arises from how procedures are captured in the registries. While we could have excluded non-revision open procedures for hips to align definitions more closely, we believe retaining this data adds valuable nuance to the results.

7. Mortality is a competing risk for reoperation. The use of Kaplan-Meier and Cox regression without competing risk analysis (e.g., Fine-Gray models) overestimates reoperation rates, particularly in T2DM patients with higher mortality.

- Authors response: Thank you for raising this methodological point. We acknowledge that mortality is a competing risk for reoperation and that methods such as Fine-Gray models can account for this. However, from a patient-centered perspective —particularly when contemplating joint replacement — it is clinically meaningful to consider outcomes that may occur during the patient's lifetime. Therefore, we believe that the use of Kaplan-Meier and Cox regression remains appropriate for addressing the research question in this context. This is supported by Ranstam et al Acta Orthop. 2011 Jun;82(3):258-67.

8. The nonsignificant association between T2DM and knee reoperation (adjusted HR 1.09, 95% CI 0.96–1.24) may reflect inadequate power rather than a true null effect. A post-hoc power calculation is needed to confirm whether the sample size was sufficient for subgroup analyses.

- Authors response: Thank you for your comment. We acknowledge the concern regarding statistical power in subgroup analysis. However, a post-hoc power calculation does not add insight beyond what is already conveyed by the confidence intervals. Instead, we rely on the observed estimates and their precision to interpret the

findings.

Reviewer: 4
Dr. Kyle Dack, University of Bristol

Comments to the Author:
Statistical review summary:

- This is very well written, and clearly reports the methodology and results. The data is high-quality with an excellent sample size, and the authors have used reliably modelling methods which minimize the risk of bias. I have only a few minor suggestions to improve the clarity, but regarding the statistical analysis I have no concerns.
    - Authors response: Thank you very much for your positive feedback. We're glad to hear that the methodology and statistical analysis were clear and robust. We appreciate your suggestions for improving clarity and will incorporate them to further strengthen the manuscript.

    - Abstract:
      Clear – no suggestions.
        - Authors response: Thank you

- Introduction:
  Line 102: A reference is made to inconsistency in prior literature, but I don't think the previous paragraph clearly showed what this inconsistency is. Is it that Lenguerrand et al did not find an association with T2DM? If so, I suggest directly reporting this because the current summary only implies it.
        - Authors response: Thank you for your thoughtful comment. We agree that the inconsistency in the literature could be more clearly articulated. To address this, we have revised the paragraph to explicitly state which publications showed no statistical association between T2DM and revision or adverse event. The text has been updated from "Previously published studies often identify diabetes to be associated with an increased risk for complications after hip or knee replacement however this varies depending on the definition of complication, size of studied cohort and definition of diabetes diagnosis. Lenguerrand et al suggest that poorer outcomes after total hip and knee replacement are associated more with obesity and other comorbidities rather than with diabetes." to "Previously published studies often identify

diabetes to be associated with an increased risk for complications after hip or knee replacement. However, there are publications suggesting no significant association between T2DM and revision or adverse events. The definition of complication, size of studied cohort and definition of diabetes diagnosis varies between studies adding to the complexity in interpreting the results. Lenguerrand et al suggest that poorer outcomes after total hip and knee replacement are associated more with obesity and other comorbidities rather than with diabetes." (row 100-105).

Methods:

- Line 115. Is it possible to report a summary of what data was taken from each database? Even if perhaps just a summary table in the supplementary materials, this could be useful for readers wanting to know more about each data source.
    - Authors response: Thank you for the helpful suggestion. We agree that providing more information of the data extracted from each register would enhance transparency and assist readers in understanding the scope and utility of the data sources. In response, we have clarified what type of data are obtained from each database, such as clinical interventions, comorbidities, demographic and socioeconomic variables, and mortality information. The text was updated from "The cohort includes data from three national quality registers (the Swedish Arthroplasty Register, the National Diabetes Register and the Swedish Osteoarthritis Register), linked with data from the National Patient Register and the Prescribed Drug Register from the National Board of Health and Welfare, the Total Population Register, the Longitudinal Integrated Database for Health Insurance and Labour Market Studies (LISA) and the Cause of Death Register from Statistics Sweden" to "The cohort was constructed by linking data from multiple national registers in Sweden. Clinical information related to treatment and interventions associated with respective diagnoses was obtained from the Swedish Arthroplasty Register, the National Diabetes Register, and the Swedish Osteoarthritis Register. Comorbidity data were extracted from the National Patient Register which contains diagnoses from specialist inpatient and outpatient care,

while the Prescribed Drug Register provided information on pharmacological treatments, used as proxies for comorbid conditions. Demographic variables were sourced from the Total Population Register, and socioeconomic indicators were retrieved from the Longitudinal Integrated Database for Health Insurance and Labour Market Studies (LISA). Finally, the Cause of Death Register from Statistics Sweden supplied data on mortality and causes of death."

- Figure 1. Very clear and helpful but I suggest increasing resolution if possible because when zoomed the text is pixelated.
    - Authors response: Thank you for your valuable feedback regarding the image resolution. We agree that the previous version was suboptimal. The image has now been updated with a higher-resolution version.

- What was the rationale for the use of different end dates for risk of reoperation, mortality, and adverse events? If it was due to differences in data availability I suggest stating this for clarity.
    - Authors response: Thank you for your comment. The use of different end dates for the outcomes—reoperation, mortality, and adverse events—was due to differences in data availability across the respective registers. This is described in the Outcome Measures section of the Methods chapter (row 168-169). No revision has been made.

- Line 174. The authors stated that duration of disease diagnosis was not available. I wonder if this could be a potential mediator which future studies could explore? E.g. if there are different associations between those with long term diabetes vs those recently diagnosed. If this is a valid idea I suggest mentioning in the discussion?
    - Authors response: Thank you for your comment. We would like to clarify that the statement in line 174 refers specifically to the timing of adverse events, not the date of diabetes diagnosis. As noted, the National Patient Registry does not contain information on the date of inpatient adverse event diagnosis, and therefore the date of hospital admission was used as a proxy for the timing of adverse events. This has been clarified in the text by changing the

text from “Adverse events were limited to inpatient care and the date of admission was used as a proxy for date of diagnosis as the National Patient Registry lacks information on the date of diagnosis.” to “Due to the absence of adverse event diagnosis date for inpatient care in the National Patient Registry the date of admission was used as a proxy for adverse event diagnosis date. “ However, we fully agree that the duration of diabetes diagnosis could be a meaningful mediator in the relationship between diabetes and the studied outcomes. Differences between individuals with long-standing versus newly diagnosed diabetes may influence risk profiles and treatment responses.

- Line 205. It is not clear from this whether the all models included mediators, or whether models with and without mediators were generated.
    - Authors response: Thank you for your comment. We acknowledge that the original wording may have been unclear regarding the inclusion of mediators in the models. This has now been clarified in the revised text to specify that reoperation, adverse event and mortality outcomes have all been assessed with and without confounders and mediators. The text has been updated with “Outcomes measures in terms of reoperation, adverse event and mortality were assessed unadjusted and adjusted for confounders, and in an explorative model also for mediators, listed in the previous section.” (row 210-212).

- Results: IQR is reported as ICQ in places, one should be used for consistency.
    - Authors response: Thank you for highlighting this inconsistency. We have reviewed the manuscript and corrected all instances where "ICQ" was used, ensuring that "IQR" is consistently applied throughout the text.

- Results are really clear and well reported, I have almost no suggestions. My only feedback is that this line: “The inclusion of the explorative mediators together with the confounding showed similar results (data not shown).” is jarring because it references results being showed, but

then data not shown. I suggest either changing it to the negative “When explorative mediators were included in the models, the results did not differ notably”, or even better to add those results as supplementary tables which can be referenced.

- Authors response: Thank you for your positive feedback and helpful suggestion. We agree that the original phrasing “The inclusion of the explorative mediators together with the confounding showed similar results (data not shown).” was potentially confusing. In response, we have revised the sentence to reflect your proposed wording: *“When explorative mediators were included in the models, the results did not differ notably.”* (row 269-270). We appreciate your careful reading and constructive input.
